# Diagnostic Ability and Capacity of Optical Coherence Tomography-Angiography to Detect Retinal and Vascular Changes in Patients with Fibromyalgia

**DOI:** 10.1155/2022/3946017

**Published:** 2022-08-27

**Authors:** Elena Garcia-Martin, Alvaro Tello, Elisa Vilades, Javier Perez-Velilla, Beatriz Cordon, Diego Fernandez-Velasco, Javier Garcia-Campayo, Marta Puebla-Guedea, Maria Satue

**Affiliations:** ^1^Ophthalmology Department, Miguel Servet University Hospital, Zaragoza, Spain; ^2^Miguel Servet Ophthalmology Research and Innovation Group (GIMSO), Aragon Institute for Health Research (IIS Aragón), University of Zaragoza, Zaragoza, Spain; ^3^University of Zaragoza, Zaragoza, Spain; ^4^Psychiatry Department, Primary Care Research Network (REDIAPP), Miguel Servet University Hospital, Zaragoza, Spain

## Abstract

**Background:**

To evaluate the neuroretina and retinal vasculature of fibromyalgia (FM) patients and calculate a linear discriminant function (LDF) to improve retinal parameters' contribution to FM diagnosis.

**Methods:**

Fifty FM patients and 232 healthy controls underwent retinal evaluation using swept-source optical coherence tomography (SS-OCT) angiography (Triton plus; Topcon) and spectral domain OCT (SD-OCT) (Spectralis; Heidelberg). The macular (m) and peripapillary (p) retinal nerve fibre layer (RNFL) and ganglion cell layer (GCL) were assessed, as was the macular vascular density. A logistic regression analysis was performed, and an LDF was calculated to evaluate OCT's contribution to FM diagnosis.

**Results:**

With Triton OCT, the patients presented pRNFL thinning in the temporal sector (*p*=0.006). Spectralis OCT measurements showed decreased pRNFL in patients in the following sectors: superonasal, *p*=0.001; nasal, *p*=0.001; inferonasal, *p*=0.006; temporal, *p*=0.001; and inferotemporal, *p*=0.001. No significant differences were observed in the macular vascular plexus between patients and controls. However, vascular density in the superior sector showed a strong inverse correlation with disease duration (*r* = −0.978, *p*=0.022). The LDF calculated for Spectralis OCT yielded an area under the ROC curve of 0.968.

**Conclusions:**

FM patients present RNFL thinning observable using SS- and SD-OCT. However, these patients show similar vascular density in the macular area to healthy controls. The LDF that combines several RNFL parameters obtained using Spectralis OCT gives this device a powerful ability to differentiate between healthy individuals and individuals with FM.

## 1. Introduction

Fibromyalgia (FM) is a pathology characterized by a constellation of symptoms that include widespread chronic musculoskeletal pain and associated fatigue, sleep disturbance, and other somatic and cognitive symptoms. For many patients, these symptoms persist for years and result in frequent recourse to health services. For a proportion of patients, fibromyalgia and its symptoms can be progressive and debilitating [1]. Fibromyalgia is also known as chronic pain syndrome or chronic fatigue syndrome and affects approximately 2% of the global population [2, 3]. In these patients, affectation on quality of life ranges from mild to severe [2–4].

Although FM's pathophysiology is not fully understood, current theories suggest that the clinical presentation of FM depends on central phenomena rather than peripheral dysfunction. Changes in brain perfusion, structure, and functional responses to pain have also been described in FM patients [5–8].

In recent years, digital imaging technologies applied in ophthalmology have provided clinicians with new biomarkers for diagnosing and monitoring neurodegenerative diseases. Optical coherence tomography (OCT) allows clinicians to measure neuroretinal layers, which are thinner in patients suffering from multiple sclerosis, Alzheimer's, and Parkinson's disease [9–12]. Although FM has been postulated as a pathology related to changes in brain function, vasculature, and structure, possible structural changes affecting the neuroretina in FM patients have barely been evaluated. Our group recently found retinal changes observable using OCT in a cohort of patients with FM [13], extending the list of neurological syndromes with a possible neurodegenerative course and adding to what is known of its pathophysiology to date.

In this study, we analyze a cohort of FM patients using different OCT technologies. Based on the emission of infrared light waves, these devices obtain a high number of one-dimensional scans (A-scans), which are performed at several depths to create a two-dimensional image (B-scan). Those B-scans, if acquired closely and rapidly, can be translated into a volumetric image. Swept-source (SS)-OCT uses a 1,050 nm centered wavelength and performs 100,000 A-scans/second (one-dimensional images), thereby obtaining high-quality images of the retina and choroid. SS-OCT Angio™ measures the superficial vascular plexus of the macula, providing information on retinal perfusion in patients evaluated using SS-OCT.

Spectralis OCT, which performs 40,000 A-scans/second and is widely used in clinical ophthalmology, was also used in our study to provide further data on the peripapillary retinal layers. Using these different OCT devices, we calculated two different linear discriminant functions (LDFs) to improve the efficacy of retinal parameters in FM diagnosis.

## 2. Methods

A total of 50 patients (50 eyes) with confirmed FM were enrolled in a cross-sectional study. All patients were sex- and age-matched with 232 healthy individuals. The study received the approval of the local ethics committee (Scientific Research Ethics Committee of Aragon: CEICA). All procedures conformed to the Declaration of Helsinki and all study participants provided informed consent in writing.

Patients were recruited from the primary care research network's FM patient study population in Zaragoza, Spain. The FM diagnosis was as per the 1990 American College of Rheumatology criteria for FM [14]. The type of FM, age at diagnosis, disease duration, and treatment were recorded by a psychiatrist specializing in FM, who evaluated the patients and was blind to the ophthalmology assessment. FM severity was evaluated using the Fibromyalgia Impact Questionnaire (FIQ), while the impact on day-to-day activities and quality of life was evaluated using the Euro Quality of Life 5D (EQ-5D) scale. The ophthalmologic evaluation consisted of anterior segment assessment, visual field test, best-corrected visual acuity (BCVA) based on the Snellen scale, OCT evaluation, and funduscopic examination. All participants were evaluated by two neuro-ophthalmologists, who were blind to the psychiatrist's evaluation. The exclusion criteria comprised patients with BCVA <0.4 (decimal, measured with the Snellen chart), significant refractive errors (>5 dioptres of spherical equivalent refraction or 3 dioptres of astigmatism), intraocular pressure ≥21 mmHg, concomitant ocular disease (including the history of glaucoma or retinal pathology), media opacifications, and systemic conditions (especially neurodegenerative processes) potentially affecting the visual system. The healthy control subjects presented no history or evidence of ocular or neurological disease (BCVA > 0.4). Each eye was assessed independently, and one eye per subject was selected at random (unless only one eye met the exclusion criteria).

### 2.1. OCT Evaluation

Retinal structure measurements were obtained using the two OCT devices employing different image acquisition/analysis systems. The SS Triton OCT (Topcon, Japan) was used to evaluate the RNFL and GCL thickness in both the peripapillary and macular areas. Spectralis spectral domain OCT (Heidelberg Engineering, Heidelberg, Germany) was utilized to assess the RNFL thickness in the peripapillary area using two different analysis protocols: RNFL-G (glaucoma) and RNFL-N (Nsite Axonal analytics).

SS Triton OCT uses a tunable laser as a light source to deliver a 1,050 nm centered wavelength, reaching a speed of 100,000 A-scans/second. This study used the 3D (H) Macula + 5 LineCross protocol (wide protocol), which expedites the evaluation of the macular + peripapillary area by analyzing a wide retinal area. This protocol employs a wide scanning range that focuses on both the macular (ETDRS scan/TSNIT scan) and peripapillary areas (TSNIT scan). Macular TSNIT analysis obtains measurements of six macular sectors (temporal superior, superior, nasal superior, temporal inferior, inferior, and nasal inferior) with automated segmentation of the RNFL and GCL. Similarly, the peripapillary TSNIT scan analyses the RNFL and GCL in six different sectors (nasal, nasal inferior, nasal superior, temporal, temporal superior, and temporal inferior).

Spectralis OCT was used to measure and display the RNFL thickness around the peripapillary area in 6 different sectors and also to calculate the average thickness. The Spectralis OCT Nsite Axonal Analytics application centres the temporal region of the scan in the viewing window to facilitate analysis of axonal loss in the papillomacular bundle (PMB). The RNFL thickness graph for the RNFL-N scans displays the results in the following order: nasal, inferior, temporal, superior, and nasal (opposite to the RNFL-G application). The PMB protocol focuses on the PMB and the nerve fiber around the macula, conducting isotropic scans of 20 different sectors. It provides extensive information about the thickness of the main bundle that transmits the stimuli captured by the macula photoreceptors.

All scans were carried out by the same experienced operator who was blind to the results of the psychiatric and ophthalmologic evaluations. An internal fixation target was used to maximize reproducibility, and OCT output did not undergo manual correction.

### 2.2. OCT-Angiography Evaluation

**Figure 1:**
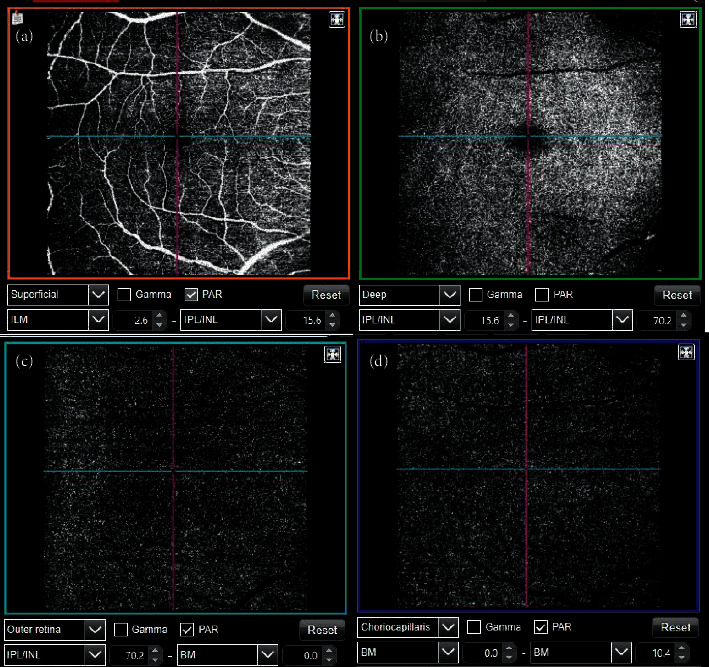
Vasculature analysis was obtained using the Triton (Topcon, Japan) SS-OCT angiography in a healthy subject included in the study. The vascular plexus can be displayed at different ocular levels: superficial (a), deep (b), outer retina (c), and choriocapillaris (d).

SS Triton OCT angiography was used to analyze the vasculature. SS-OCT Angio™ images were obtained using a 6 × 6 mm cube centered on the fovea at a resolution of 320 × 320. Proprietary Topcon IMAGEnet® (version 1.19) software was used to measure surface area after automatically segmenting the macular area into superficial vascular plexuses (SVP, SVP-foveal avascular zone) and including large vessels. Vessel density was calculated from the internal limiting membrane to the inner plexiform layer based on the percentage of the scanned area (6 × 6 mm) occupied by vessel lumens (Figure 1).

### 2.3. Fibromyalgia Evaluation

Each FM patient also completed the FIQ and EQ-5D questionnaires on disease impact, quality of life, and day-to-day activities. The validated Spanish version of these questionnaires was used [15, 16], in the case of the FIQ, assigning a score from 0–100. The higher the FIQ score, the greater the disease impact. The EQ-5D contains five questions (in three response categories) addressing the dimensions of mobility, self-care, usual activities, pain, and anxiety/depression. EQ-5D scores are assigned on a scale of 0–100, with 100 being the best possible patient health status and 0 being the worst possible health status [17].

### 2.4. Statistical Analysis

To ensure accurate image assessment, only images with a quality score of >55/100 and >25/40 were selected for retinal analysis with Triton and Spectralis OCT, respectively; for OCTA evaluation, the image quality threshold was >40/100. Poor-quality scans were discarded and, in such cases, image acquisition was repeated. One eye per patient was selected at random for the study.

All variables were entered into a database created with commercially available software (FileMaker Pro 8.5; File-Maker, Inc., Santa Clara, CA), with the modifier variables being age and intraocular pressure. Statistical analysis was performed with commercially available predictive analytics software (SPSS, version 20.0; SPSS, Inc., Chicago, IL). Sample distribution normality was confirmed by the Kolmogorov–Smirnov test. Patients and controls were compared using the Student's *t*-test (two-tailed), and a *p* value ≤0.05 was considered statistically significant for all calculations. In addition, the Bonferroni correction was applied to multiple comparisons (see Tables).

Logistic regression analysis was carried out to ascertain whether any of the structural parameters evaluated in the study had the potential to differentiate between healthy and FM eyes. Our dataset underwent two binary logistic regression analyses (one using only data from Triton OCT, and one using only data from Spectralis OCT), in which FM was the dependent variable (yes or no), and vascular density (the latter only for Triton OCT) and the retinal measurements in each analyzed sector were the predictive variables. The relative importance of each independent variable was evaluated using the stepwise binary logistic regression analysis following the forward Wald method. An LDF was obtained from regression analysis for each OCT device, calculating a score based on the weighted sum of the predictive variables. The receiver operating characteristic (ROC) curves were plotted for all parameters and compared against the proposed LDF.

Possible associations between structural and vascular changes and disease duration were analyzed using Spearman's correlation test.

## 3. Results

This cross-sectional study analyzed 50 eyes from 50 FM patients and 232 eyes from 232 control subjects. There were no significant differences in age, sex, or intraocular pressure between the groups. The FM phenotype distribution was as follows: biological FM, 10 patients (20%); depressive FM, 12 patients (24%); and atypical FM, 28 patients (56%). The FIQ mean score was 62.21 ± 17.27 and the EQ-5D mean score was 43.89 ± 17.14 (all demographic variables and significances are presented in Table 1).

### 3.1. Structural Measurements

Significant differences between FM patients and control subjects were mostly observed using Spectralis OCT. With Triton OCT, patients presented significantly reduced RNFL thickness in the temporal sector of the peripapillary area (70.53 ± 10.77 microns (±standard deviation) in patients vs. 77.21 ± 10.77 microns (±standard deviation) in controls, *p*=0.006) (Table 2). The macular GCL was thinner in FM patients (superotemporal: 68.24 ± 8.25 vs. 71.99 ± 7.91, *p*=0.026; inferonasal: 70.71 ± 9.35 vs. 75.15 ± 10.08, *p*=0.029), although these values did not reach Bonferroni significance (Table 3).

Using Triton OCT, strong inverse correlations were observed between disease duration and peripapillary GCL thickness (nasal: *r* = −1.00, *p* < 0.001) and macular GCL thickness (superior: *r* = −0.996, *p*=0.004; superonasal: *r* = −0.999, *p*=0.001; inferonasal: *r* = −0.982, *p*=0.018).

Spectralis OCT measurements (RNFL-G application) showed decreased peripapillary RNFL in FM patients affecting the superonasal (*p*=0.001), nasal (*p*=0.001), inferonasal (*p*=0.006), temporal (*p*=0.001), and inferotemporal (*p*=0.001) sectors (Table 4). The RNFL-N analysis also revealed a significantly reduced temporal sector in FM patients (*p*=0.003). The PMB was thinner in patients with FM, although Bonferroni significance was not reached (*p*=0.019) (Table 4). A strong inverse correlation was observed between disease duration and RNFL thickness in the nasal sector (glaucoma application) (*r* = −0.975, *p*=0.025).

### 3.2. Vasculature Analysis

No significant differences were observed between FM patients and control subjects as regards superficial vascular plexus density in the macular area (Table 5). The density observed in the superior sector showed a strong inverse correlation with disease duration (*r* = −0.978, *p*=0.022).

### 3.3. Logistic Regression Analysis

**Figure 2:**
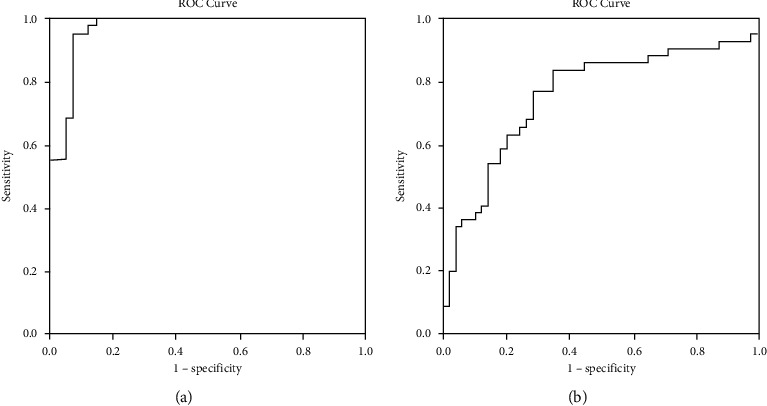
(a) Area under the ROC curve corresponding to the Spectralis LDF, using only retinal parameters, for diagnosis of FM. (b) Area under the ROC curve corresponding to the Triton LDF, for the diagnosis of FM.

The structural Spectralis OCT and Triton OCT parameters that accounted for the greatest amount of error were identified using a stepwise procedure and were added to the model. This procedure was repeated progressively for each next-best variable. All 768 Spectralis OCT A-scans measuring RNFL thickness were included in the statistical analysis to calculate the Spectralis (S)-LDF, which was defined as follows: 33.099 − (0.075 × PMB) − (0.166 × superotemporal RNFL-N) − (0.301 × temporal RNFL-N) − (0.092 × inferotemporal RNFL-N) − (0.195 × inferonasal RNFL-N) − (0.303 × nasal RNFL-N) − (0.189 × superonasal RNFL-N) + (0.924 × average RNFL-N) − (0.093 × superotemporal RNFL-G) − (0.137 × superior RNFL-G) − (0.390 × inferotemporal RNFL-G) − (0.124 × inferonasal RNFL-G) − (0.246 × nasal RNFL-G) − (0.483 × superonasal RNFL-G) + (1.468 × average RNFL-G). The calculated area under the ROC was 0.968 (Figure 2(a)).

All the data provided by Triton OCT (RNFL scans, GCL scans, and vascular density parameters) were used to calculate the Triton (T)-LDF. This was repeated for the Spectralis data. The T-LDF was defined as −9.102 + (0.434 × inferior mRNFL) + (0.088 × nasal pRNFL) − (0.220 × temporal pRNFL). The calculated area under the ROC was 0.758 (Figure 2(b)).

## 4. Discussion

This study assessed retinal structure and vasculature in FM patients. To the best of our knowledge, this is the first study to evaluate the ability of SS-OCT (A) to detect retinal anomalies in these patients. Additionally, for the first time, an LDF for OCT measurements is provided that offers a high potential for diagnosis of FM.

Our patients showed a significant decrease in the peripapillary RNFL thickness compared to healthy control subjects, and this decrease was detectable with both Triton OCT and Spectralis OCT, though it was the latter that identified the most significantly affected sectors. To our knowledge, there are no published studies on axonal loss in FM other than our previous findings in a different cohort of patients [16, 18]. These papers also identified the RNFL loss in the peripapillary area using the Spectralis and Cirrus OCT. Contrary to our previously observed results, Triton OCT did not detect significant changes in the macular area in these patients, despite an observed decrease in macular GCL thickness. The lack of significant macular differences between patients and control subjects measured with the Triton OCT may be due to the smaller sample size in the current study. Notwithstanding the above, structural measurements provided by Triton OCT presented the strongest and most numerous correlations with disease duration. This result supports previous data that showed that the GCL is correlated with disease severity progression [18], suggesting that the GCL is indeed a potential biomarker for FM progression. More studies on retinal changes in FM patients are undoubtedly needed to corroborate our findings.

A significant decrease in regional cerebral blood flow in certain brain areas has been observed in FM patients and is related to pain perception [5]. Research on retinal vascular perfusion in FM patients is likewise practically nonexistent [19, 20]. Bambo et al. used colorimetric analysis software to evaluate perfusion at the optic nerve head in FM patients, observing that hemoglobin levels were decreased in those patients, especially within the neuroretinal rim. The macular area, however, was not assessed. Ulusoy et al. [20] provided new insights into the pathophysiology of this syndrome by detecting choroidal thinning in the macular area in FM patients. They suggested that this decrease in blood perfusion may be related to alterations in autonomic nervous system function. In this study, and for the first time, we used SS-OCTA to evaluate the retinal vasculature of the macular area in FM patients. No changes versus control subjects were observed. Our results add new information on vascular involvement in FM and indicate that macular vasculature does not appear to be affected in these patients and might not be responsible for the observed retinal changes.

Current views of the aetiology of FM suggest the involvement of central phenomena, with the central nervous system playing a leading role [21]. Abnormalities in sensory signalling, including changes in key neurotransmitters and a reduction in descending control, have also been found to be associated with central sensitization in these patients [22]. FM patients may also have altered pain pathways that result in abnormal pain amplification [23, 24] and a chronic proinflammatory state (in both the CNS and peripheral tissues). However, there are not many hypotheses to explain retinal thinning in FM. Earlier studies have described neurobiological and structural brain irregularities in these subjects [2, 8]. Our findings support this theory and suggest that neurodegeneration is causing observed RNFL depletion and contributing to the pathology of FM.

The most significant finding in our study is the calculational ability of OCT devices to contribute to FM diagnosis. To date, several studies have provided LDFs using OCT measurements in the diagnosis of other neurodegenerative processes, such as multiple sclerosis [25] and Parkinson's disease [26]. The current LDF calculated for the Triton OCT yielded an area under the ROC curve of 0.758. However, the Spectralis OCT yielded an area under the ROC curve of 0.968, which is higher than any of the LDFs previously calculated for other diseases. This suggests that Spectralis OCT provides high diagnostic accuracy and makes a powerful contribution to FM diagnosis.

There are some limitations to this study. First, despite a clear trend towards a decrease in the macular GCL in some sectors, we could not demonstrate significant differences using Bonferroni's correction for multiple comparisons. Since our past research using the Cirrus OCT did reveal the GCL thinning in these patients, we believe our sample was probably too small for the Triton OCT to detect significant differences in this cohort.

Additionally, we did not find any other published studies on retinal changes and FM other than our own work, meaning our results cannot be supported by previous findings by external researchers. We are unsure why there are no other published papers. One of the reasons could be the lack of positive results. Another might be the lack of previous publications on the relationship between FM and retinal measurements, which might discourage new research on this topic. However, such a situation would, however, be highly counterproductive for this kind of research since more results are needed to support (or contradict) new findings, not only for our group but for science in general.

To conclude, FM patients present decreased RNFL thickness observable by SS- and SD-OCT. Patients with FM show similar vascular density levels in the macular area when measured using the SS-OCTA as healthy control subjects, suggesting retinal structural changes are not due to retinal hypoperfusion and supporting the hypothesis of neurodegeneration in FM. Based on the area under the ROC curve, the LDF that combines several RNFL parameters obtained with Spectralis OCT gives this device a powerful ability to differentiate between healthy individuals and individuals with FM. We believe that further study, with larger sample sizes, is of great importance, especially as regards the assessment of treatment effectiveness and study of the pathophysiology of this disease.

## Figures and Tables

**Table 1:** Demographic parameters of patients with fibromyalgia and healthy controls included in the study.

Demographic parameter	FM (*N* = 50)	Controls (*N* = 249)	*P*
Male/female (%)	8/92	9/91	0.822
Age	57.92 ± 8.08	57.31 ± 12.33	0.737
IOP	15 ± 2.01	14 ± 2.36	0.574
Disease duration	16.88 ± 6.47		

FM subtype			
Depressive	12 (24%)		
Biological	10 (20%)		
Atypical	28 (56%)		

FM severity			
FIQ	62.21 ± 17.27		
EQ-5D	43.89 ± 17.14		

FM, fibromyalgia; IOP, intraocular pressure; FIQ, fibromyalgia impact questionnaire; EQ-5D, Euro Quality of Life 5D.

**Table 2:** Peripapillary measurements (in microns) of the retinal nerve fiber layer and the ganglion cell layer in patients with fibromyalgia and healthy controls using swept-source Triton optical coherence tomography. Bold numbers indicate *p* < 0.05, the asterisk indicates significant values according to Bonferroni corrections (*p* < 0.007).

	Sector	FM (mean ± SD)	Controls (mean ± SD)	*p*
pRNFL	Temporal	70.53 ± 10.77	77.21 ± 10.77	**0.006** ^*∗*^
	Superotemporal	133.61 ± 19.12	141.15 ± 19.53	0.059
	Inferotemporal	141.23 ± 20.34	149.41 ± 22.18	0.064
	Superonasal	114.03 ± 28.96	114.93 ± 26.65	0.875
	Nasal	91.12 ± 18.89	87.87 ± 13.86	0.332
	Inferonasal	136.81 ± 33.29	133.50 ± 28.10	0.596

pGCL	Temporal	56.85 ± 9.40	53.66 ± 5.93	**0.045**
	Superotemporal	43.91 ± 10.95	40.85 ± 6.64	0.096
	Inferotemporal	41.50 ± 6.98	39.81 ± 6.10	0.201
	Superonasal	42.73 ± 9.42	41.64 ± 6.30	0.495
	Inferonasal	37.07 ± 6.10	38.16 ± 5.25	0.352
	Nasal	38.91 ± 5.68	39.37 ± 3.99	0.646

FM, fibromyalgia; SD, standard deviation; pRNFL, peripapillary retinal nerve fiber layer; pGCL, peripapillary ganglion cell layer.

**Table 3:** Macular structural analysis (in microns) of the retinal nerve fiber layer and the ganglion cell layer in patients with fibromyalgia and healthy subjects using swept-source Triton optical coherence tomography. Bold numbers indicate *p* < 0.05. The significance according to the Bonferroni's correction was calculated as *p* < 0.007.

	Macular sector	FM (mean ± SD)	Controls (mean ± SD)	*p*
mRNFL	Superotemporal	25.06 ± 4.12	24.88 ± 2.00	0.779
	Superior	38.49 ± 5.39	39.00 ± 4.94	0.624
	Superonasal	45.67 ± 6.64	47.10 ± 5.25	0.245
	Inferonasal	70.71 ± 9.35	75.15 ± 10.08	0.563
	Inferior	40.74 ± 8.32	39.82 ± 4.67	0.500
	Inferotemporal	26.17 ± 2.73	26.19 ± 2.15	0.976

mGCL	Superotemporal	68.24 ± 8.25	71.99 ± 7.91	**0.026**
	Superior	70.39 ± 6.42	72.57 ± 8.50	0.168
	Superonasal	73.29 ± 7.24	76.53 ± 9.30	0.064
	Inferonasal	70.71 ± 9.35	75.15 ± 10.08	**0.029**
	Inferior	67.38 ± 6.71	70.28 ± 8.23	0.066
	Inferotemporal	70.41 ± 8.33	73.73 ± 8.12	0.053

FM, fibromyalgia; SD, standard deviation; mRNFL, macular retinal nerve fiber layer; mGCL, macular ganglion cell layer.

**Table 4:** Peripapillary measurements (in microns) of the retinal nerve fiber layer in patients with fibromyalgia and healthy controls using the two different protocols for spectralis OCT—the RNFL-G protocol and the RNFL-Axonal analytics protocol. Bold numbers indicate *p* < 0.05, asterisks indicate significant values according to the Bonferroni's correction for multiple comparisons (*p* < 0.007).

Spectralis	Sector	FM (mean ± SD)	Controls (mean ± SD)	
RNFL-G	Mean	98.60 ± 11.96	101.54 ± 11.40	0.185
	Superonasal	67.13 ± 13.27	95.51 ± 22.71	**0.001** ^*∗*^
	Nasal	131.82 ± 20.98	98.69 ± 34.73	**0.001** ^*∗*^
	Inferonasal	139.11 ± 22.12	124.74 ± 29.66	**0.006** ^*∗*^
	Temporal	80.63 ± 18.64	123.93 ± 36.34	**0.001** ^*∗*^
	Inferotemporal	107.94 ± 27.67	88.68 ± 28.92	**0.001** ^*∗*^
	Superotemporal	118.15 ± 30.73	130.24 ± 21.90	**0.014**

RNFL axonal analytics	Mean	97.69 ± 10.98	104.17 ± 12.05	**0.012**
	Nasal	80.48 ± 19.47	86.07 ± 18.73	0.178
	Superonasal	110.34 ± 27.37	110.82 ± 25.92	0.934
	Inferonasal	116.10 ± 29.73	120.22 ± 26.74	0.495
	Inferotemporal	136.26 ± 20.36	146.39 ± 21.29	**0.025**
	Temporal	65.74 ± 15.91	74.83 ± 12.16	**0.003** ^*∗*^
	Superotemporal	128.31 ± 19.71	137.37 ± 17.66	**0.025**
	PMB	51.19 ± 13.34	57.04 ± 9.39	**0.019**

FM, fibromyalgia; SD, standard deviation; RNFL-G, retinal nerve fiber layer-glaucoma protocol; PMB, papillomacular bundle.

**Table 5:** Vascular plexus density in the macular area measured with the Triton optical coherence tomography angiography in patients with fibromyalgia and healthy controls.

Sector	FM (mean ± SD)	Controls (mean ± SD)	*p*
Central	21.19 ± 4.11	21.41 ± 4.35	0.788
Nasal	45.36 ± 2.20	46.20 ± 3.66	0.165
Inferior	48.21 ± 3.00	49.43 ± 3.92	0.073
Temporal	46.12 ± 2.46	46.84 ± 3.74	0.250
Superior	49.27 ± 2.00	48.93 ± 3.33	0.538

FM, fibromyalgia; SD, standard deviation.

## Data Availability

Data supporting the conclusions will be provided by the corresponding author upon reasonable request.

## References

[1] Bair M. J., Krebs E. E. (2020). Fibromyalgia. *Annals of Internal Medicine*.

[2] Clauw D. J. (2014). Fibromyalgia: a clinical review. *JAMA*.

[3] Janssens K. A. M., Zijlema W. L., Joustra M. L., Rosmalen J. G. M. (2015). Mood and anxiety disorders in chronic fatigue síndrome, fibromyalgia, and irritable bowel syndrome: results from the LifeLines cohort study. *Psychosomatic Medicine*.

[4] Wolfe F., Clauw D. J., Fitzcharles M. A. (2010). The American college of rheumatology preliminary diagnostic criteria for fibromyalgia and measurement of symptom severity. *Arthritis Care & Research*.

[5] Mountz J. M., Bradley L. A., Modell J. G. (1995). Fibromyalgia in women. *Arthritis & Rheumatism*.

[6] Kwiatek R., Barnden L., Tedman R. (2000). Regional cerebral blood flow in fibromyalgia: single-photon-emission computed tomography evidence of reduction in the pontine tegmentum and thalami. *Arthritis & Rheumatism*.

[7] Gracely R. H., Petzke F., Wolf J. M., Clauw D. J. (2002). Functional magnetic resonance imaging evidence of augmented pain processing in fibromyalgia. *Arthritis & Rheumatism*.

[8] Jensen K. B., Srinivasan P., Spaeth R. (2013). Overlapping structural and functional brain changes in patients with long-term exposure to fibromyalgia pain. *Arthritis & Rheumatism*.

[9] Garcia-Martin E., Rodriguez-Mena D., Herrero R. (2013). Neuro-ophthalmologic evaluation, quality of life, and functional disability in patients with MS. *Neurology*.

[10] Ratchford J. N., Quigg M. E., Conger A. (2009). Optical coherence tomography helps differentiate neuromyelitis optica and MS optic neuropathies. *Neurology*.

[11] Satue M., Seral M., Otin S. (2014). Retinal thinning and correlation with functional disability in patients with Parkinson’s disease. *British Journal of Ophthalmology*.

[12] Polo V., Garcia-Martin E., Bambo M. P. (2014). Reliability and validity of cirrus and spectralis optical coherence tomography for detecting retinal atrophy in Alzheimer’s disease. *Eye*.

[13] Garcia-Martin E., Garcia-Campayo J., Puebla-Guedea M. (2016). Fibromyalgia is correlated with retinal nerve fiber layer thinning. *PLoS One*.

[14] Wolfe F., Smythe H. A., Yunus M. B. (1990). The American college of rheumatology 1990 criteria for the classification of fibromyalgia: report of the multicenter criteria committee. *Arthritis & Rheumatism*.

[15] Rivera J., Gonzalez T. (2004). The fibromyalgia impact questionnaire: a validated Spanish version to assess the health status in women with fibromyalgia. *Clinical & Experimental Rheumatology*.

[16] Badía X., Roset M., Montserrat S., Herdman M., Segura A. (1999). The Spanish version of EuroQol: a description and its applications. European quality of life scale. *Medical Clinics of North America*.

[17] Kind P., Dolan P., Gudex C., Williams A. (1998). Variations in population health status: results from a United Kingdom national questionnaire survey. *BMJ*.

[19] Pilar Bambo M., Garcia-Martin E., Gutierrez-Ruiz F. (2015). Study of perfusion changes in the optic disc of patients with fibromyalgia syndrome using new colorimetric analysis software. *Journal Français d’Ophtalmologie*.

[20] Ulusoy M. O., Kal A., Işik-Ulusoy S., Kal O. (2018). Choroidal thickness in patients with fibromyalgia and correlation with disease severity. *Indian Journal of Ophthalmology*.

[21] Sawaddiruk P., Paiboonworachat S., Chattipakorn N., Chattipakorn S. C. (2017). Alterations of brain activity in fibromyalgia patients. *Journal of Clinical Neuroscience*.

[22] Finnerup N. B., Haroutounian S., Kamerman P. (2016). Neuropathic pain: an updated grading system for research and clinical practice. *Pain*.

[23] Häuser W., Ablin J., Fitzcharles M. A. (2015). Fibromyalgia. *Nature Reviews Disease Primers*.

[24] Sluka K. A., Clauw D. J. (2016). Neurobiology of fibromyalgia and chronic widespread pain. *Neuroscience*.

[25] Garcia-Martin E., Pablo L. E., Herrero R. (2012). Diagnostic ability of a linear discriminant function for spectral-domain optical coherence tomography in patients with multiple sclerosis. *Ophthalmology*.

[26] Garcia-Martin E., Satue M., Otin S. (2014). Retina measurements for diagnosis of Parkinson disease. *Retina*.

